# *JCPP Advances* in 2025: A landmark year for growth, quality, and visibility

**DOI:** 10.1002/jcv2.70036

**Published:** 2025-08-10

**Authors:** Henrik Larsson

**Affiliations:** ^1^ School of Medical Sciences Örebro University Örebro Sweden

**Keywords:** child and adolescent mental health, impact factor, open science, registered reports

## Abstract

In this editorial, we reflect on a milestone year for *JCPP Advances*, marked by our first Journal Impact Factor and significant growth in submissions, readership, and citations. We highlight expanded editorial expertise, strengthened commitments to open science, and new initiatives such as Registered Reports. Recent indexing across PsycINFO, PubMed, Scopus, and Web of Science enhances our global visibility. The September 2025 issue exemplifies our dedication to rigorous, impactful research, including evidence syntheses, participatory studies, and methodological innovation. Together, these developments position *JCPP Advances* as a leading open‐access platform advancing child and adolescent mental health research worldwide.

**Key points**

- Milestone Year: JCPP Advances achieved its first Journal Impact Factor (3.1), placing it in the top quartile of psychology journals, alongside major indexing milestones in PsycINFO, PubMed, Scopus, and Web of Science.
- Sustained Growth: Significant increases in full‐text views, submissions, and citations reflect the journal’s expanding global reach and influence.
- Commitment to Quality: Editorial board expansion, adoption of Registered Reports, and ongoing promotion of open science reinforce methodological rigour and transparency.
- September Issue Spotlight: Fifteen diverse papers highlight innovation and relevance, with featured articles on evidence synthesis, participatory research, and measurement validation.

## RECOGNITION AND GROWTH

A couple of years ago, I wrote an editorial titled ‘*What a first year for JCPP Advances—Time to celebrate!*’ Today, I am delighted to write a follow‐up—this time with even more reason to celebrate. In 2025, we received our first Journal Impact Factor, and the results are nothing short of remarkable. With a 2‐year Impact Factor of 3.1 and a 5‐year Impact Factor of 3.5, *JCPP Advances* is officially ranked in the top quartile (Q1) among psychology journals. This is an extraordinary milestone for a young journal and a testament to the high‐quality work of our authors, the rigorous evaluation by our editors and reviewers, and the enthusiastic support of the international child and adolescent mental health community.

But this is not just a year of recognition—it has also been a year of continued growth. Our Full Text Views increased from 117,626 to 167,254 when comparing Q1–Q2 2024 to Q1–Q2 2025, showing a strong rise in global engagement. Submissions also rose from 55 to 75 in the same period, reflecting the growing trust in *JCPP Advances* as a platform for impactful research. In terms of citations, we had 319 2‐year citations in 2024, and we are already at 253 citations so far in 2025, suggesting a continued trajectory of influence.

## ADVANCING QUALITY AND OPEN SCIENCE

In parallel with the journal's growing reach and influence, we have also expanded our editorial board to meet increasing demands and to cover new, emerging areas of research. Over the past year, we have welcomed Dr Jessie Baldwin, Dr Alessio Bellato, Dr Laurie Hannigan, Dr Giorgia Michelini, and Dr Simone Pisano to the team of Joint Editors. These strategic recruitments reflect our commitment to supporting the journal's growth—both in volume and scope. These accomplished and highly engaged researchers bring deep expertise across several critical areas—including evidence synthesis (e.g., systematic reviews, meta‐analyses, umbrella reviews, and meta‐research), as well as cognitive neuroscience, treatment studies, and open science and reproducible research practices. Their addition allows *JCPP Advances* to better support authors working at the forefront of these domains and to strengthen the methodological and conceptual rigour of the journal.

Our editorial team—all active researchers themselves—are deeply committed to fostering a constructive and supportive peer review culture. They understand the challenges of publishing in today's academic environment and go the extra step to provide timely, thoughtful, and constructive feedback to authors. This culture is central to our mission: to offer a high‐quality, open‐access platform that promotes excellence and innovation in child and adolescent mental health research.

Since the first issue in 2021, *JCPP Advances* has been committed to advancing open science and reproducible research practices. We have implemented several initiatives, including mandatory data availability statements and the offering of open science badges, to promote transparency and integrity in research. We have also curated special issues with a dedicated focus on *open science and reproducible research*, helping to highlight important trends and foster community dialogue. Our 2024 special issue on participatory research marked a significant step forward. Participatory approaches—which actively involve stakeholders such as patients, families, and communities throughout the research process—represent a vital component of open science. They ensure that studies are not only scientifically rigorous, but also relevant, ethical, and impactful (MacLeod et al., 2024).

Several editorials and editorial perspectives published in *JCPP Advances* have contributed to a broader understanding of open and reproducible research practices. For example, Larsson et al. (2025) emphasised the value of preregistration of high‐quality protocols, while Fabiano et al. (2024) underscored how improving meta‐research literacy can enhance the quality, transparency, and reproducibility of both primary research and meta‐research outputs.

Most recently, *JCPP Advances* launched Registered Reports as a new article format—an important step toward enhancing research transparency and combating publication bias (Baldwin & Larsson, 2024). In a Registered Report, the study protocol is peer‐reviewed and accepted before the research is conducted. If the protocol is judged to be of high quality and the research question important, *JCPP Advances* commits to publishing the study regardless of its results. This approach helps ensure that publication decisions are based on rigour and relevance, not on whether findings are positive, negative, or null. By adopting Registered Reports, we aim to support more credible science and provide a clear incentive for researchers to pre‐register their methods and analysis plans.

Another important set of developments is the growing recognition of *JCPP Advances* across major scholarly indexing platforms—a key marker of visibility and quality. In the past 2 years, the journal has achieved several significant milestones: it was indexed in PsycINFO and PubMed in 2023, followed by inclusion in Scopus and the Web of Science in 2024. These achievements greatly enhance the discoverability of our published work and ensure that authors' contributions are accessible to a broad, global readership. They also reflect the high editorial standards and methodological rigour that underpin our commitment to advancing the science of child and adolescent mental health.

These accomplishments are the result of a collective effort. I would like to extend heartfelt thanks to our authors, reviewers, editors, readers, and international partners who make this possible. *JCPP Advances* is truly becoming a global hub for high‐quality, open‐access research in child psychology and psychiatry.

## HIGHLIGHTS FROM THE SEPTEMBER 2025 ISSUE

The September issue of *JCPP Advances* brings together a high‐quality collection of 15 open‐access papers that reflect the journal's overarching goals: to promote rigorous, relevant, and impactful research in child and adolescent mental health. The issue spans diverse topics, study designs, and global cohorts—showcasing both methodological innovation and clinical relevance. Three papers exemplify the issue's breadth, quality, and relevance—spanning evidence synthesis, participatory research, and measurement validation in large‐scale cohorts.

In a timely and policy‐relevant systematic review and meta‐analysis, Mughal et al. (2025) examined the impact of therapeutic interventions (TIs) on mortality following adolescent self‐harm. While the pooled analysis found no statistically significant effect of TIs on mortality, the review demonstrated a reduction in repeated self‐harm and suicide attempts, reinforcing the clinical value of TIs. Importantly, these findings must be interpreted with caution: the total number of deaths across included trials was very low (*n* = 21), which limits the stability of mortality estimates and means the findings should be viewed as suggestive rather than definitive. This paper exemplifies the type of high‐quality evidence‐synthesis that is of special interest to *JCPP Advances*. Our journal has made a clear commitment to publishing rigorous, transparent systematic reviews that inform clinical care and policy. The editorial board includes members with substantial expertise in systematic reviews, meta‐analyses, and meta‐research, who are dedicated to upholding the highest methodological standards in evidence synthesis. This aligns with our broader ambition to support decision‐making through the best available evidence.

Barclay et al. (2025) investigated factors contributing to earlier recognition of Attention‐Deficit/Hyperactivity Disorder (ADHD) and why some children remain undiagnosed despite showing symptoms and functional impairment. The study found that children with more emotional and behavioural difficulties, greater emotional dysregulation, lower cognitive ability, and poorer prosocial skills were more likely to receive an earlier diagnosis. Recognised cases were also more likely to have parental depression or anxiety, lower physical activity, and a co‐occurring autism diagnosis. Importantly, the study was shaped through consultation with a Youth Advisory Group of young people with lived experience of neurodevelopmental conditions. Their input informed the research questions and comparisons, ensuring the study addressed real‐world priorities from the perspective of those most affected. This integration of lived experience reflects an important and growing movement in child and adolescent mental health research toward more participatory and inclusive science. Sex‐specific analyses revealed that emotional dysregulation was a key differentiating factor between recognised and unrecognised ADHD in males, but not females—highlighting the need for gender‐sensitive diagnostic practices.

Zelenina et al. (2025) addressed a fundamental question for researchers working with large‐scale longitudinal cohorts: how valid are commonly used measures of adolescent depression? The study focused on the Child Behavior Checklist's DSM‐5‐Oriented Affective Problems scale (CBCL‐Aff)—the only continuous measure of depression available in the widely used Adolescent Brain Cognitive Development (ABCD) study. Using data from ABCD, as well as two independent cohorts—the Healthy Brain Network and the Brazilian High Risk Cohort Study—the study found that CBCL‐Aff showed strong predictive validity when compared with parent‐ and clinician‐reported diagnoses, but not with child self‐reports, highlighting the challenge of informant discrepancies in youth mental health research. The approach taken by Zelenina and colleagues is ambitious, drawing on three independent datasets, each with its own strengths and limitations, to provide a robust validation across contexts. By critically evaluating the psychometric performance of CBCL‐Aff, the study helps establish an essential foundation for meaningful research using large cohort datasets. As developmental science increasingly depends on open‐access longitudinal data, psychometrically sound measures are key to producing reliable and interpretable findings.

## LOOKING AHEAD

As we look ahead, *JCPP Advances* will continue to lead the way in promoting open, rigorous, and inclusive science—with the goal of improving outcomes for children, adolescents, and young people worldwide. The achievements we have celebrated this year—from our first Journal Impact Factor to expanded indexing, editorial growth, and methodological innovation—reflect the collective dedication of our authors, editors, reviewers, and readers. They also reaffirm our commitment to providing a trusted platform for high‐quality, accessible research that informs both science and practice in child and adolescent mental health. We remain ambitious about the journal's future and optimistic about the role it can play in supporting a global research community driven by transparency, collaboration, and real‐world impact. Thank you for being part of this journey—we look forward to continuing it with you.

## AUTHOR CONTRIBUTIONS

**Henrik Larsson:** Conceptualization; writing—original draft; writing—review and editing.

## CONFLICT OF INTEREST STATEMENT

Henrik Larsson reports receiving grants from TAKEDA and Shire Pharmaceuticals; personal fees from and serving as a speaker for Medice, Shire/Takeda Pharmaceuticals and Evolan Pharma AB; all outside the submitted work. Henrik Larsson is editor‐in‐chief of *JCPP Advanc*es.

## ETHICAL CONSIDERATIONS

Not applicable.

## Data Availability

Data sharing not applicable to this article as no datasets were generated or analysed.

## References

[jcv270036-bib-0001] Baldwin, J. R. , & Larsson, H. (2024). Implementing open science and reproducible research practices in mental health research through registered reports. JCPP Advances, 4(3), e12275. 10.1002/jcv2.12275 39411475 PMC11472803

[jcv270036-bib-0002] Barclay, I. , Sayal, K. , Ford, T. , John, A. , Taylor, M. J. , Thapar, A. , Langley, K. , & Martin, J. (2025). Investigating the reasons behind a later or missed diagnosis of attention‐deficit/hyperactivity disorder in young people: A population cohort study. JCPP Advances, e12301. 10.1002/jcv2.12301 PMC12446718 40979729

[jcv270036-bib-0003] Fabiano, N. , Gupta, A. , Fiedorowicz, J. G. , & Solmi, M. (2024). On the value of meta‐research for early career researchers: A commentary. JCPP Advances, 4(2), e12235. 10.1002/jcv2.12235 38827987 PMC11143950

[jcv270036-bib-0004] Larsson, H. , Chang, Z. , & Man, K. K. C. (2025). Preregistration of high‐quality protocols in pharmacoepidemiology research. JCPP Advances, e70020. 10.1002/jcv2.70020 PMC12698272 41395335

[jcv270036-bib-0005] MacLeod, A. , Manitsa, I. , & De Brito, S. (2024). Participatory research in child psychology & psychiatry: Embracing untidiness to break new ground. JCPP Advances, 4(4), e12284. 10.1002/jcv2.12284 39734927 PMC11669778

[jcv270036-bib-0006] Mughal, F. , Young, P. , Stahl, D. , Asarnow, J. R. , & Ougrin, D. (2025). Mortality in adolescents after therapeutic intervention for self‐harm: A systematic review and meta‐analysis. JCPP Advances, e12302. 10.1002/jcv2.12302 PMC7617579 40224150

[jcv270036-bib-0007] Zelenina, M. , Pine, D. S. , Stringaris, A. , & Nielson, D. M. (2025). Validation of CBCL depression scores of adolescents in three independent datasets. JCPP Advances, e12298. 10.1002/jcv2.12298 PMC12446721 40979734

